# Investigations on the cytotoxicity and antimicrobial activities of terezine E and 14-hydroxyterezine D

**DOI:** 10.1590/1414-431X2023e12404

**Published:** 2023-04-07

**Authors:** M. Mojally, R. Abdou, W. Bokhari, S. Sab, M. Dawoud, A. Albohy

**Affiliations:** 1 Department of Pharmaceutical Chemistry, Faculty of Pharmacy, Umm Al-Qura University, Makkah, Saudi Arabia; 2 Department of Pharmacognosy, Faculty of Pharmacy, Umm Al-Qura University, Makkah, Saudi Arabia; 3 Department of Pharmacognosy, Faculty of Pharmacy, Helwan University, Cairo, Egypt; 4 Department of Applied Biochemistry, Faculty of Science, University of Jeddah, Jeddah, Saudi Arabia; 5 Faculty of Pharmacy, King Abdulaziz University, Jeddah, Saudi Arabia; 6 Department of Pharmaceutics, Faculty of Pharmacy, Umm Al-Qura University, Makkah, Saudi Arabia; 7 Department of Pharmaceutical Chemistry, Faculty of Pharmacy, The British University in Egypt, El-Sherouk City, Cairo, Egypt; 8 Center for Drug Research and Development (CDRD), Faculty of Pharmacy, The British University in Egypt, El-Sherouk City, Cairo, Egypt

**Keywords:** Terezine E, 14-Hydroxyterezine D, Cytotoxicity, Antimicrobial activity, Molecular docking

## Abstract

Secondary metabolites produced by endophytes are an excellent source of biologically active compounds. The newly isolated natural products terezine E and 14-hydroxyterezine D are endophytic metabolites exhibiting anticancer activity recently identified by our team (https://doi.org/10.1080/14786419.2018.1489393). In our current study, we evaluated their affinity for binding to the active site of histone deacetylase (PDB ID: 4CBT) and matrix metalloproteinase 9 (PDB ID: 4H3X) by molecular docking using AutoDock Vina software after having tested their cytotoxic activities on three cell lines (human ductal breast epithelial tumor cells (T47D)-HCC1937), human hepatocarcinoma cell line (HepG2)-HB8065), and human colorectal carcinoma cells (HCT-116)-TCP1006, purchased from ATCC, USA)). Additionally, their antimicrobial activities were investigated, and their minimum inhibitory concentration (MIC) values were determined against *P. notatum* and *S. aureus* by the broth microdilution method. Higher cytotoxicity was observed for terezine E against all tested cell lines compared to 14-hydroxyterezine D. Molecular docking results supported the high cytotoxicity of terezine E and showed higher binding affinity with 4CBT with an energy score of 9 kcal/mol. Terezine E showed higher antibacterial and antifungal activities than 14-hydroxyrerezine D: MIC values were 15.45 and 21.73 µg/mL against *S. aureus* and 8.61 and 11.54 µg/mL against *P. notatum*, respectively.

## Introduction

**Figure 1:**
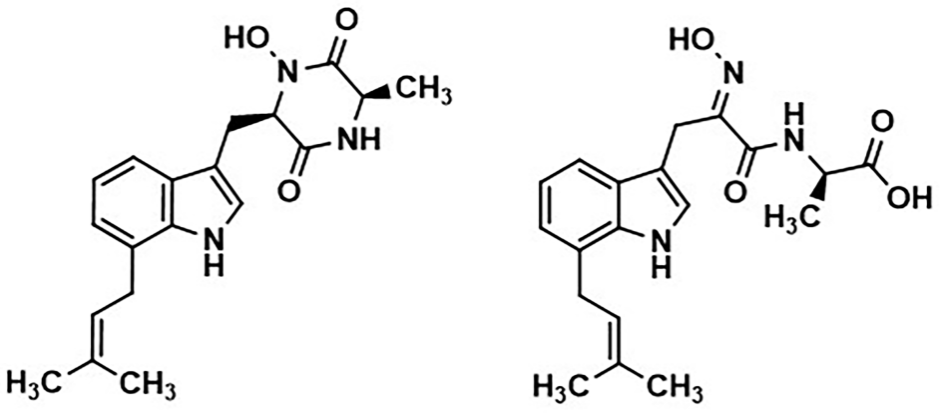
Chemical structures of 14-hydroxyterezine D and terezine E.

The microscopic species known as endophytes inhabit inter- and intracellular spaces of tissues of advanced plants without causing harmful effects to their host plants. They have been recognized as a rich source of bioactive natural products (1). Investigation of the medicinal plant *Centaurea stoebe* for its endophytes (2) resulted in the isolation of a bioactive mucor species from which terezine E and 14-hydroxyterezine D (Figure 1) were isolated as new natural products (3). Both natural products exerted cytotoxic effects against the HeLa cell line and antiproliferative activities against HUVEC and K-562 cancer cell lines. Cytostatic effects were exhibited by both natural products, with 14-hydroxyterezine D showing higher antiproliferative activity and terezine E exerting higher cytotoxicity. Furthermore, the isolated compounds were tested for antifungal activity against *A. terreus*, and terezine E exhibited higher activity than 14-hydroxyterezine D (3) (minimal inhibitory concentration (MIC) 43.6 µg/mL for 14-hydroxyterezine D and 39.7 µg/mL for terezine E) compared with the positive control nystatin (2), which had a MIC value of 15.63 µg/mL.

Molecular docking is an *in silico* structure-based method extensively used in drug discovery, which can highlight the therapeutic interest of novel compounds or natural products (4). Molecular docking depends on the information derived from the 3D structure of a target of interest and on the ranking databases of molecules according to the complementary electron and the structure of ligands to the target protein. Molecular docking is accomplished first by molecular orientation of a ligand within a receptor. Second, the complementary ligand is estimated through the use of a scoring function (5,6).

In this study, we were interested in a deeper investigation of the biological activities of terezine E and 14-hydroxyterezine. Therefore, they were tested for antimicrobial activity against several bacterial and fungal test strains. Since colorectal cancer has been reported to be the most prevalent type of cancer in Asia (7), while breast cancer is considered the most common malignancy among women in Saudi Arabia (8) and hepatocellular carcinoma is a major worldwide health concern (9), we were interested in examining the cytotoxicity of terezine E and 14-hydroxyterezine on human ductal breast epithelial tumor cells (T47D), human hepatocarcinoma cell line (HepG2), and human colorectal carcinoma cells (HCT-116) using the MTT assay. In addition, evaluation of the potential binding affinity of terezine E and 14-hydroxyterezine D to the active site of histone deacetylase enzyme (PDB ID: 4CBT) and matrix metalloproteinase 9 (PDB ID: 4H3X) was also studied using molecular docking.

## Material and Methods

### Antimicrobial activity

Terezine E and 14-hydroxyterezine D were tested against several bacterial (*Escherichia coli* ATCC 25922, *Bacillus subtilis* ATCC 6633, *Staphylococcus aureus* ATCC 25923) and fungal (*Aspergillus terreus* ATCC 74135, *Penicillium notatum ATCC* 9478, *Penicillium chrysogenum* ATCC 10106) test strains in an agar diffusion assay using nystatin (1 µg/mL) and ciprofloxacin (5 µg/mL) as positive controls for antifungal and antibacterial activities, respectively. The minimum inhibitory concentration (MIC) of each compound against the test strains *P. notatum* and *S. aureus* was determined by the broth microdilution method according to the literature (10-13).

### Cytotoxic assay

Terezine E and 14-hydroxyterezine D were tested for cytotoxicity against three cancer cell lines (human ductal breast epithelial tumor cells (T47D)-HCC1937, human hepatocarcinoma cell line (HepG2)-HB8065, and human colorectal carcinoma cells (HCT-116)-TCP1006 purchased from ATCC, USA) using the MTT assay. Dulbecco's modified Eagle's medium (DMEM) was used to culture HepG2 and T47D cells. McCoy's 5a (modified) medium was used for HCT-116 cells, which were enriched with 100 units/mL streptomycin sulfate, 10% fetal bovine serum, 250 ng/mL amphotericin B, and 2 mM L-glutamine containing 100 units/mL penicillin G sodium. Cells were kept at 37°C in humidified air (5% CO_2_) at sub-confluence. After trypsin/EDTA treatment at 37°C, monolayer cells were harvested for sub-culturing. The cells were used when confluence reached 75%. Samples under investigation were dissolved in dimethyl sulfoxide (DMSO) and diluted one thousand times. All chemicals were obtained from Sigma-Aldrich (USA), while cell culture materials were purchased from Cambrex BioScience (Denmark). Experiments were repeated four times. The cytotoxic effect was evaluated against HepG2, T-47D, and HCT-116 cells with the MTT (3-[4,5-dimethylthiazole-2-yl]-2,5-diphenyltetrazolium bromide) cell viability assay according to the literature (14,15), which is based on the ability of active mitochondrial dehydrogenase enzyme of active cells to cleave the tetrazolium rings of the yellow MTT and produce a dark blue insoluble formazan crystal. The extent of MTT reduction was quantified as previously described (14) by measuring the absorbance at 570 nm. A 96-well microplate was employed to plate the cells (0.5×10^5^ cells/well) in serum-free media and cells were treated with different concentrations of each compound for 24 h at 37°C, in a humidified atmosphere with 5% CO_2_. The media were separated after incubation and 40 µL MTT solution/well was added and incubated for an additional 4 h. The MTT crystals were solubilized by adding 180 µL of acidified isopropanol/well, and the plate was agitated at room temperature, followed by determination of the absorbance at 570 nm using a microplate ELISA reader (Lonza, USA]. The percentage of relative viability was determined according to the literature (14,16).

### Molecular docking

The structures of compounds used in this study were drawn in Discovery Studio Visualizer (BIOVIA v. 2021, Dassault Systemes BIOVIA; https://www.3ds.com) and saved as SDF format, and the energy of the ligand minimized and converted to PDBQT file using PyRx (17). The 3-dimentional crystal structure of histone deacetylase (PDB ID: 4CBT) and matrix metalloproteinase 9 (PDB ID: 4H3X) were retrieved from the protein data bank database in PDB format (https://www.rcb.org/). Proteins were combined with inhibitors: histone deacetylase inhibitor is (1R,2R,3R)-2-[4-(5-fluoranylpyrimidin-2-yl)phenyl]-N-oxidanyl-3-phenyl-cyclopropane-1-carboxamide and matrix metalloproteinase 9 inhibitor is N-2-(biphenyl-4-ylsulfonyl)-N-2-(isopropyloxy)-acetohydroxamic acid. The macromolecules were prepared using chain A of histone deacetylase (PDB ID: 4CBT) and chain A along with zinc ions for matrix metalloproteinase 9 (PDB ID: 4H3X). The inhibitor, water molecules, and other heteroatoms were deleted from the protein, and polar hydrogen atoms were added using Discovery Studio software v. 2021 and then saved as prepared proteins files in PDB format for further analysis. For histone deacetylase, docking was done in a grid box centered on the co-crystalized ligand with the following x, y, and z dimensions; 14.4643, 9.8022, and 9.3913 Å, respectively. For matrix metalloproteinase 9, the grid box dimensions used were 7.4149, 6.9369, and 13.7158 Å in the x, y, and z dimensions, respectively, centered on co-crystalized ligand. Docking studies on our target proteins were also performed for panobinostat, which is reported to play a significant therapeutic role in targeting aggressive triple-negative breast cancer cell types (18), inhibiting hepatocellular carcinoma cell lines (19,20) and is a pan-deacetylase inhibitor (21). Docking was done using Autodock Vina (22) using exhaustiveness of 16. Validation of docking procedure was done through the redocking of co-crystalized ligands in their corresponding proteins followed by calculation of RMSD between the crystal and docked structures using DockRMSD (23). RMSD of less than two angstroms was considered acceptable. Test compounds were then docked using the same procedure.

## Results and Discussion

### Investigation of antimicrobial activity

The minimum inhibitory concentration (MIC) of terezine E and 14-hydroxyterezine D against the test strains *P. notatum* and *S. aureus* were determined by the broth microdilution method according to the literature (11,12,24). MIC values of 15.45 and 20.73 µg/mL against *S. aureus* and 8.61 and 11.54 µg/mL against *P. notatum* were exhibited by terezine E and 14-hydroxyrerezine D, respectively (Table 1). The reference nystatin exerted a MIC value of 15.63 µg/mL and ciprofloxacin 0.43 µg/mL. Their MIC values were also examined against the plant pathogen *Fusarium oxysporum* and results revealed a MIC value of 54 µg/mL for terezine E and 65.81 µg/mL for 14-hydroxyterezine D compared to the positive standard amphotericin B, which showed a MIC value of 2.39 µg/mL against *F. oxysporum*.

**Table 1:** Minimum inhibitory concentration (MIC) values of the antifungal and antibacterial activities of terezine E and 14-hydroxyterezine D against *Staphylococcus aureus, Fusarium oxysporum*, and *Penicillium notatum* using nystatin, ciprofloxacin, and amphotericin B as positive controls.

	MIC (µg/mL)		
	*S. aureus*	*F. oxysporum*	*P. notatum*
Terezine E	15.45	54.00	8.61
14-Hydroxyterezine D	21.73	65.81	11.54
Nystatin	-	-	15.64
Ciprofloxacin	0.43	-	-
Amphotericin B	-	2.39	-

### Investigation of cytotoxic activity

**Figure 2:**
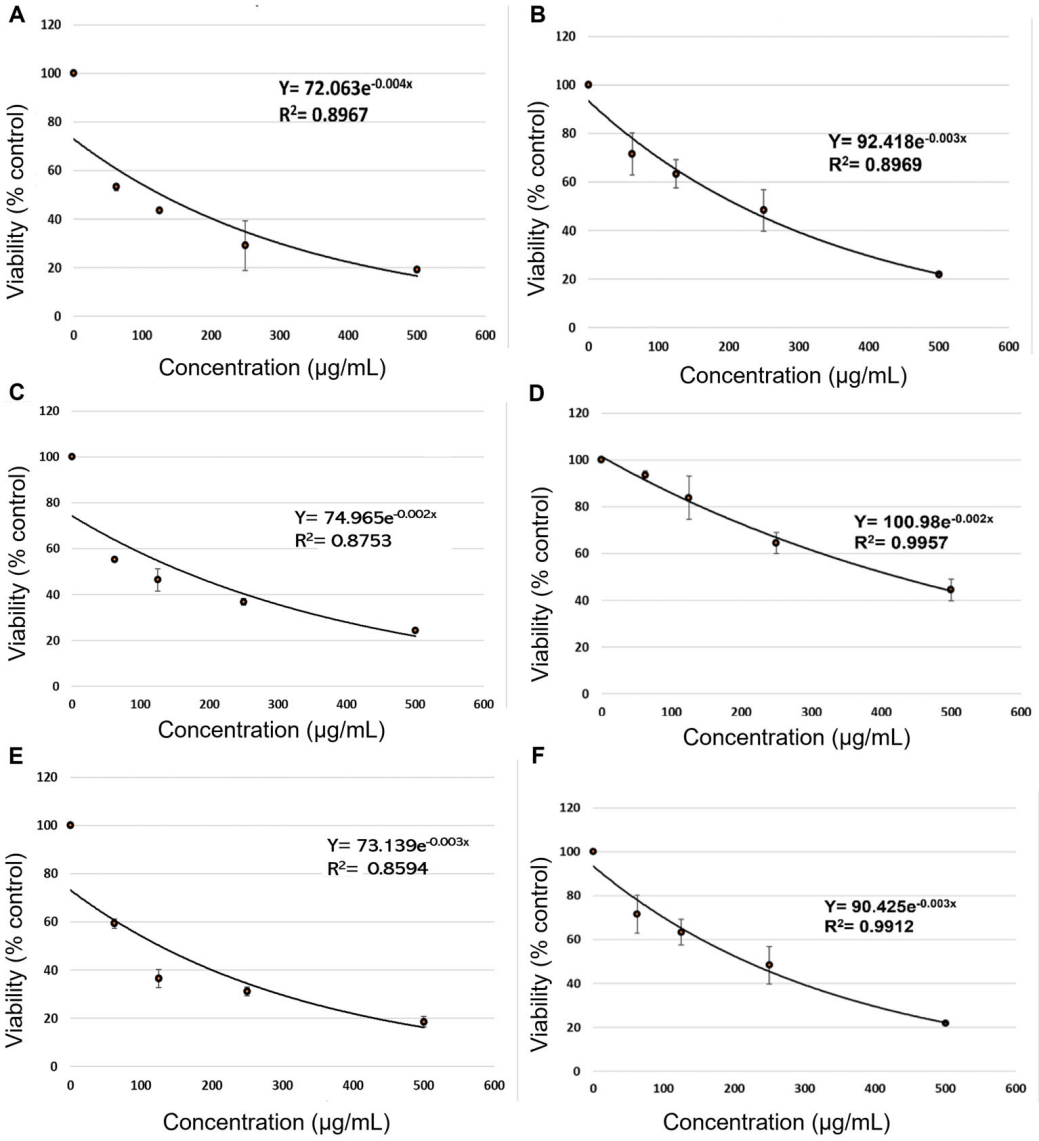
**A**, Cytotoxicity of terezine E against Hep-G2 cell line. **B**, Cytotoxicity of 14-hydroxyterezine D against Hep-G2 cell line. **C**, Cytotoxicity of terezine E against T47-D cell line. **D**, Cytotoxicity of 14-hydroxyterezine D against T47-D cell line. **E**, Cytotoxicity of terezine E against HCT-116 cell line. **F**, Cytotoxicity of 14-hydroxyterezine D against HCT-116 cell line. Results are reported as means±SE of at least three independent experiments (3 replications each). Student’s *t*-test was used for statistical analyses.

The cytotoxicity of terezine E and 14-hydroxyterezine D was tested against three cancer cell lines (human ductal breast epithelial tumor cells (T47D), human hepatocarcinoma cell line (HepG2), and human colorectal carcinoma cells (HCT-116) using the MTT assay. The cytotoxic effect was evaluated by employing the MTT (3-[4,5-dimethylthiazole-2-yl]-2,5-diphenyltetrazolium bromide) cell viability assay according to the literature (14,15), which is based on the ability of mitochondrial dehydrogenase to cleave tetrazolium rings of the yellow MTT and produce blue insoluble formazan crystals. The extent of MTT reduction was quantified as described previously (14) by measuring the absorbance at 570 nm. Accordingly, the effect of terezine E and 14-hydroxyterezine D on the proliferation of the three cancer cell lines was studied after 24 h of incubation. Different degrees of cytotoxicity were exerted by each compound on each cell line. Results revealed high cytotoxicity of terezine E against all tested cell lines. IC_50_ values of 40, 43, and 42 µg/mL were observed for it against Hep-G2, T-47D, and HCT-116 cell line, respectively. 14-Hydroxyterezine D, on the other hand, exerted rather weak cytotoxicity by exhibiting IC_50_ values of 195, 425, and 190 µg/mL against Hep-G2, T-47D, and HCT-116 cell lines, respectively (Figure 2).

### Molecular docking

The validation of the docking protocol was done by redocking of the co-crystallized ligands for both proteins: histone deacetylase (PDB ID: 4CBT) and matrix metalloproteinase 9 (PDB ID: 4H3X). For histone deacetylase, the root-mean-square deviation (RMSD) between the crystal and docked ligands was 0.82 Å, indicating the validity of the docking protocol. A similar value (0.80 Å) was also obtained for the other protein (matrix metalloproteinase 9).

Docking of the test compounds is reported as binding energies (kcal/mol) (Table 2). Results obtained from the docking study of our target compounds, 14-hydroxyterezine D and terezene E, demonstrated that both compounds had similar docking scores against histone deacetylase, which was weaker compared to its co-crystalized ligand. When docked in the active site of matrix metalloproteinase 9, 14-hydroxyterezine D showed a docking score similar to that of terezine E and was similar to the co-crystalized ligand.

**Table 2:** Results of the docking study.

Compounds	Docking score (kcal/mol)	
	Histone deacetylase (4CBT)	Matrix metalloproteinase 9 (4H3X)
14-Hydroxyterezine D	-8.9	-8.7
Terezine E	-9.0	-7.7
Co-crystalized ligand	-10.8	-8.7
Panobinostat	-8.4	-6.5

**Figure 3:**
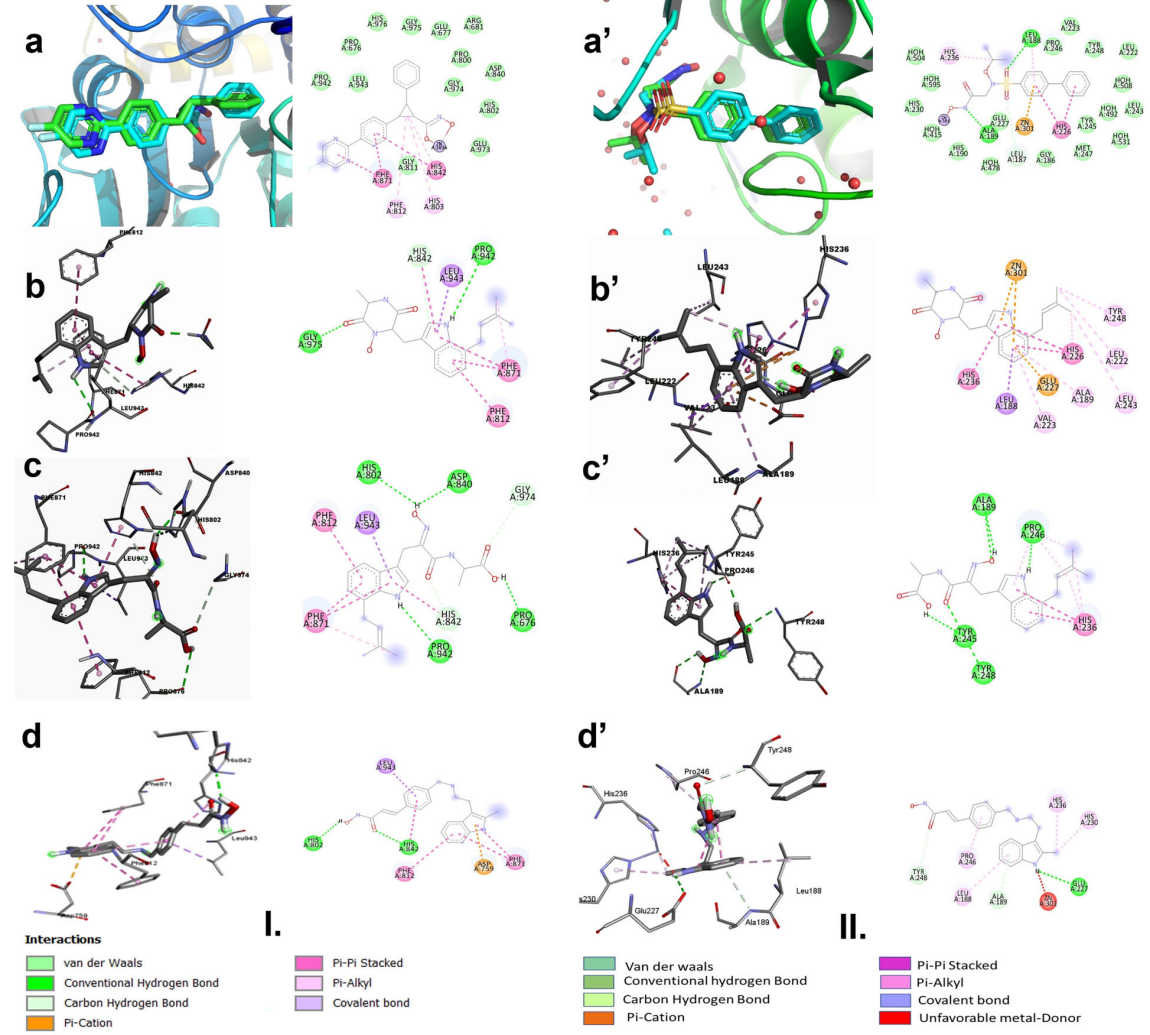
I: Docking results of our compounds against histone deacetylase (4CBT). **a**, Validation; crystal (green), docked (blue). **b**, Compound 1 docking pose and interactions. **c**, Compound 2 docking pose and interactions. **d**, Panobinostat docking pose and interactions. **II**: Docking results of our compounds against matrix metalloproteinase 9 (4H3X). **a**', Validation; crystal (green), docked (blue). **b**', Compound 1 docking pose and interactions. **c**', Compound 2 docking pose and interactions. **d**', Panobinostat docking pose and interactions.

The docking results of the target compound 1 (14-hydroxyterezine D) in histone deacetylase crystal structure protein (Figure 3) showed some similar interactions between the co-crystalized ligand and similar amino acids in the active site of the protein. The aromatic ring in compound 1 interacted with two amino acids in the active site, PHE:812 and PHE:871, by pi-pi stacked bond, and two aromatic rings in the co-crystalized ligand interacted with PHE:871 by pi-pi stacked bond. The co-crystalized ligand and compound 1 interacted with His:842 in the active site of the protein with two different types of interactions; the aromatic ring in the co-crystalized ligand interacted by pi-pi stacked bond and the pyrrolidine ring in compound 1 interacted by Van der Waals bond.

The docking results of compound 2 (terezine E) in histone deacetylase crystal structure protein (Figure 3) showed similar interactions between the co-crystalized ligand and similar amino acids in the active site of the protein. The indole ring in compound 2 interacted with PHE:871 by pi-pi stacked bond and the same type of interaction was obtained by two aromatic rings in the co-crystalized ligand with the same amino acid in the active site. The co-crystalized ligand interacted with PHE:812 by pi-alkyl bond while the aromatic ring in the target compound 2 interacted by pi-pi stacked bond with the same amino acid in the active site.

The docking results of 14-hydroxyterezine D in matrix metalloproteinase 9 crystal structure protein (Figure 3) showed also similar interactions between the co-crystalized ligand and similar amino acids in the active site of the protein. Interestingly, compound 1 coordinated with zinc atom (ZN:301) by pi-cation bond as the co-crystalized ligand of the protein. Indole ring in compound 1 interacted with HIS:226 by pi-pi stacked bond, which is the same interaction obtained with the two aromatic rings in the co-crystalized ligand with the same amino acid in the active site of the protein. The amino acid HIS:236 in the active site of the protein interacted with compound 1 and the co-crystalized ligand had a different type of interaction with a different group. The aliphatic side chain in the co-crystalized ligand interacted by pi-alkyl bond with HIS:236, while the pyrrolidine ring in compound 1 interacted by pi-pi stacked bond with HIS:236.

The indole ring in compound 2 interacted in the active site of the matrix metalloproteinase 9 (Figure 3) with HIS:236 by pi-pi stacked bond and the aliphatic side chain in the co-crystalized ligand interacted with the same amino acid by pi-alkyl bond.

Several studies proved that panobinostat has a therapeutic role in targeting aggressive triple-negative breast cancer cell types (18) and is a pan-deacetylase inhibitor (21). In addition, panobinostat is an effective inhibitor for hepatocellular carcinoma cell lines (19,20). Docking studies for panobinostat in our target proteins showed promising results, and similar interactions were obtained between panobinostat and the target proteins 14-hydroxyterezine D and terezine E. The affinity scores for docking results of panobinostat in histone deacetylase (PDB ID: 4CBT) and matrix metalloproteinase 9 (PDB ID: 4H3X) are shown in Table 2. Interestingly, docking studies of panobinostat in the target protein histone deacetylase (PDB ID: 4CBT) showed that the bicyclic system in panobinostat interacted by pi-pi stacked bond with the amino acid PHE 871. Our target compounds 14-hydroxyterezine D and terezine E also showed similar interactions with the same amino acid in the binding site of the protein (Table 2). The docking studies of panobinostat in the target protein matrix metalloproteinase 9 (PDB ID: 4H3X) showed that the aromatic ring of the bicyclic system in the compound interacted by pi-alkyl bond with the amino acid (LEU 188) in the active site of the protein. Similarly, the aromatic ring of the bicyclic system of 14-hydroxyterezine D interacted with the amino acid LEU 188 in the active site of the protein by covalent bonding.

Thus, the molecular docking carried out in this study for terezine E and 14-hydroxyterezine D on the target enzymes showed interactions similar to panobinostat with the active site of the protein. Furthermore, results of the molecular docking study supported the high cytotoxicity of the compounds under investigation, especially terezine E. Additionally, terezine E showed potent antibacterial activity against *S. aureus* (MIC=15.45 µg/mL) and antifungal activity against *P. notatum* (MIC=8.61 µg/mL). The antimicrobial activity exerted by terezine E and 14-hydroxyterezine D against the plant pathogen *F. oxysporum* suggested potential protection provided by these endophytic metabolites to their host plant, which is common in plant-endophyte symbiotic relationships. The potent cytotoxicity observed by these compounds against the tested cell lines and supported by the molecular docking study indicates their therapeutic potential and suggests performance of further studies in this respect on these compounds.

### Conclusions

The isolated endophytic metabolites terezine E and 14-hydroxyterezine D showed promising results. Terezine E demonstrated higher cytotoxicity against the tested cell lines than 14-hydroxyterezine D. Molecular docking for the target compounds terezine E and 14-hydroxyterezine D on the target enzymes histone deacetylase and matrix metalloproteinase 9 supported the high cytotoxicity results of terezine E by showing high binding affinity with histone deacetylase with an energy score of -9 kcal/mol. Terezine E also showed higher antibacterial and antifungal activities than 14-hydroxyrerezine D.

## References

[1] Newman DJ, Cragg GM (2020). Natural products as sources of new drugs over the nearly four decades from 01/1981 to 09/2019. J Nat Prod.

[2] Abdou R (2014). Bioactive metabolites from an endophyte of the medicinal plant *Centaurea stoebe*. World J Pharm Res.

[3] Abdou R, Shabana S, Rateb ME (2020). Terezine E, bioactive prenylated tryptophan analogue from an endophyte of *Centaurea stoebe*. Nat Prod Res.

[4] Pinzi L, Rastelli G (2019). Molecular docking: shifting paradigms in drug discovery. Int J Mol Sci.

[5] Dasiram JD, Gupta D (2016). Introduction to methods for molecular docking and HT virtual screening. Int J Engineering.

[6] Agarwal S, Mehrotra R (2016). An overview of molecular simulation. JSM Chem.

[7] Wong MC, Ding H, Wang J, Chan PS, Huang J (2019). Prevalence and risk factors of colorectal cancer in Asia. Intest Res.

[8] Abusanad A (2022). Breast cancer stage migration in Saudi Arabia: examining the influence of screening. Global J Quality Safety Healthcare.

[9] Poustchi H, Sepanlou S, Esmaili S, Mehrabi N, Ansarymoghadam A (2010). Hepatocellular carcinoma in the world and the middle East. Middle East J Dig Dis.

[10] Alexander BD (2002). Prophylaxis of invasive mycoses in solid organ transplantation. Curr Opin Infect Dis.

[B11] Wang Q, Bowling NA, Eschleman KJ (2010). A meta-analytic examination of work and general locus of control. J Appl Psychol.

[B12] Jorgensen JH, Ferraro MJ (2009). Antimicrobial susceptibility testing: a review of general principles and contemporary practices. Clin Infect Dis.

[13] Medjahed F, Merouane A, Saadi A, Bader A, Cioni PL, Flamini G (2016). Chemical profile and antifungal potential of essential oils from leaves and flowers of Salvia algeriensis (Desf.): a comparative study. Chilean J Agric Res.

[14] Hansen MB, Nielsen SE, Berg K (1989). Re-examination and further development of a precise and rapid dye method for measuring cell growth/cell kill. J Immunol Methods.

[15] Chahrour O, Abdalla A, Lam F, Midgley C, Wang S (2011). Synthesis and biological evaluation of benzyl styrylsulfonyl derivatives as potent anticancer mitotic inhibitors. Bioorg Med Chem Lett.

[16] Aly AA, Brown AB, Ramadan M, Gamal-Eldeen AM, Abdel-Aziz M, Abuo-Rahma GEDAA (2010). Thieno[2,3-d]pyrimidines in the synthesis of antitumor and antioxidant agents. Arch Pharm (Weinheim).

[17] Dallakyan S, Olson AJ (2015). Small-molecule library screening by docking with PyRx. Methods Mol Biol.

[18] Tate CR, Rhodes LV, Segar HC, Driver JL, Pounder FN, Burow ME (2012). Targeting triple-negative breast cancer cells with the histone deacetylase inhibitor panobinostat. Breast Cancer Res.

[19] Zopf S, Ocker M, Neureiter D, Alinger B, Gahr S, Neurath M (2012). Inhibition of DNA methyltransferase activity and expression by treatment with the pan-deacetylase inhibitor panobinostat in hepatocellular carcinoma cell lines. BMC Cancer.

[20] Lachenmayer A, Toffanin S, Cabellos L, Alsinet C, Hoshida Y, Villanueva A (2012). Combination therapy for hepatocellular carcinoma: additive preclinical efficacy of the HDAC inhibitor panobinostat with sorafenib. J Hepatol.

[21] Anne M, Sammartino D, Barginear MF, Budman D (2013). Profile of panobinostat and its potential for treatment in solid tumors: an update. OncoTargets Ther.

[22] Trott O, Olson AJ (2010). AutoDock Vina: improving the speed and accuracy of docking with a new scoring function, efficient optimization, and multithreading. J Comput Chem.

[23] Bell EW, Zhang Y (2019). DockRMSD: an open-source tool for atom mapping and RMSD calculation of symmetric molecules through graph isomorphism. J Cheminform.

[24] Jensen MB, Bjerrum PJ, Jessen TE, Nielsen JE, Joensen UN, Olesen IA (2011). Vitamin D is positively associated with sperm motility and increases intracellular calcium in human spermatozoa. Hum Reprod.

